# Vimentin, a Novel NF-κB Regulator, Is Required for Meningitic *Escherichia coli* K1-Induced Pathogen Invasion and PMN Transmigration across the Blood-Brain Barrier

**DOI:** 10.1371/journal.pone.0162641

**Published:** 2016-09-22

**Authors:** Sheng-He Huang, Feng Chi, Liang Peng, Tao Bo, Bao Zhang, Li-Qun Liu, Xuedong Wu, Nirit Mor-Vaknin, David M. Markovitz, Hong Cao, Yan-Hong Zhou

**Affiliations:** 1 Hubei Bioinformatics and Molecular Imaging Key Laboratory, College of Life Science and Technology, Huazhong University of Science and Technology, Wuhan, China; 2 Saban Research Institute of Childrens Hospital Los Angeles, Department of Pediatrics, University of Southern California, Los Angeles, California, United States of America; 3 Department of Microbiology, School of Public Health and Tropocal Medicine, Southern Medical University, Guangzhou 510515, China; 4 Department of Pathology, Southern California Research Center for ALPD and Cirrhosis, the Keck School of Medicine, University of Southern California, Los Angeles, California, United States of America; 5 Department of Clinic Laboratory, the Second Affiliated Hospital of Guangzhou Medical University, Guangzhou 510260, China; 6 Department of Pediatrics, the Second Xiangya Hospital, Central South University, Changsha, China; 7 Department of Pediatrics, Nanfang Hospital, Southern Medical University, Guangzhou 510515, China; 8 Department of Internal Medicine, Division of Infectious Diseases, 5220 MSRB III, 1150 West Medical Center Drive, University of Michigan, Ann Arbor, MI, United States of America; Shanghai Medical College, Fudan University, CHINA

## Abstract

**Background:**

NF-κB activation, pathogen invasion, polymorphonuclear leukocytes (PMN) transmigration (PMNT) across the blood-brain barrier (BBB) are the pathogenic triad hallmark features of bacterial meningitis, but the mechanisms underlying these events remain largely unknown. Vimentin, which is a novel NF-κB regulator, is the primary receptor for the major *Escherichia coli* K1 virulence factor IbeA that contributes to the pathogenesis of neonatal bacterial sepsis and meningitis (NSM). We have previously shown that IbeA-induced NF-κB signaling through its primary receptor vimentin as well as its co-receptor PTB-associated splicing factor (PSF) is required for pathogen penetration and leukocyte transmigration across the BBB. This is the first in vivo study to demonstrate how vimentin and related factors contributed to the pathogenic triad of bacterial meningitis.

**Methodology/Principal Findings:**

The role of vimentin in IbeA^+^ *E*. *coli* K1-induced NF-κB activation, pathogen invasion, leukocyte transmigration across the BBB has now been demonstrated by using vimentin knockout (KO) mice. In the in vivo studies presented here, IbeA-induced NF-κB activation, *E*. *coli* K1 invasion and polymorphonuclear neutrophil (PMN) transmigration across the BBB were significantly reduced in Vim-/- mice. Decreased neuronal injury in the hippocampal dentate gyrus was observed in Vim-/- mice with meningitis. The major inflammatory regulator α7 nAChR and several signaling molecules contributing to NF-κB activation (p65 and p-CamKII) were significantly reduced in the brain tissues of the Vim-/- mice with *E*. *coli* meningitis. Furthermore, Vim KO resulted in significant reduction in neuronal injury and in α7 nAChR-mediated calcium signaling.

**Conclusion/Significance:**

Vimentin, a novel NF-κB regulator, plays a detrimental role in the host defense against meningitic infection by modulating the NF-κB signaling pathway to increase pathogen invasion, PMN recruitment, BBB permeability and neuronal inflammation. Our findings provide the first evidence for Vim-dependent mechanisms underlying the pathogenic triad of bacterial meningitis.

## Introduction

Vimentin (Vim) contributes to IbeA-induced pathogenicities in neonatal sepsis and meningitis (NSM), which remains a major cause of death in newborns, especially in low-birth weight infants [1–9]. *E*. *coli* is the most common gram-negative bacteria causing NSM. Several *E*. *coli* virulence factors, including *ibeA*, *ibeB*, *ibeC*, *aslA*, *fim*, *traJ* and *ompA*, have been identified and characterized [1–6, 10]. Among these genes, the *ibeA* locus is able to modulate expression of other virulence factors (e.g., *aatA*, *fim*, *ibeB*, *ompA* and biofilm-associated genes) and predominantly contributes to *E*. *coli* K1-caused early-onset human NSM by inducing both pathogen penetration and PMN transmigration (PMNT) across the blood-brain barrier (BBB), which consists mainly of brain microvascular endothelial cells (BMEC) [11–13]. This locus has been used as a genetic marker in genotyping clinical *E*. *coli* strains [14]. IbeA is a unique *E*. *coli* K1 virulence factor, which is positively associated with multidrug resistance (MDR), and significantly more prevalent in the *E*. *coli* strains causing early infections (0–3 days after birth) in human NSM [12, 15, 16]. The *ibeA* locus is also prevalent in avian *E*. *coli* strains, suggesting that poultry may be a vehicle for human *E*. *coli* pathogens and those avian strains may potentially serve as a reservoir of virulence genes for meningitic strains [17–18]. The *IbeA* locus was initially identified as a genetic determinant contributing to *E*. *coli* invasion of the BBB, one of the hallmark features of this disease [4, 10,19].

The IbeA-human BMEC (HBMEC) surface protein interaction and subsequently induced signal transduction were shown to be essential for bacterial invasion [6, 20, 21]. Our previous studies have demonstrated that the specific interaction between IbeA and its primary receptor vimentin is the upstream signaling event that is required for the caveolae/lipid raft (LR)-dependent entry of *E*. *coli* K1 into HBMECs [6, 21]. Subsequently, the co-receptor PSF and related signaling molecules (e.g., ERK, caveolin-1, α7 nAChR) are recruited to the LR microdomains [6, 20, 22]. Bacterial invasion was positively correlated with phosphorylation of Vim at serine 82 by CaM kinase II (CaMKII), IbeA^+^ *E*. *coli*-induced phosphorylation of Akt [5], and Erk [6]. IbeA^+^ *E*. *coli* and IbeA-coated beads induced the clustering of Vim that was correlated with increased entry of bacteria and beads. These studies suggest that Vim-mediated signaling is essential for IbeA^+^ *E*. *coli* K1 invasion. Vim has emerged as an important organizer of a number of critical proteins that contribute to attachment, migration, LR-mediated cell signaling [23–24]. It is also involved with bacterial infections caused by Group A streptococci, Salmonella and *P*. *multocida* [21,25]. It has been shown that caveolae/LRs are signaling platforms for IbeA-induced pathogenicities [6]. Caveolae/LRs are becoming increasingly recognized as significant entry portals for the endocytosis of a wide variety of microbial pathogens and toxins [26]. Recently, we have shown that caveolae/LRs are essential for *C*. *neoformans* penetration across the BBB [27]. OmpA (outer membrane protein A)^+^ *E*. *coli* K1 entry into HBMEC was shown to be clathrin-independent but caveolae-dependent [28]. Caveolae, however, are not specifically associated with specific virulence factors in those studies, as OmpA is a major outer membrane protein present in both pathogenic *E*. *coli* K1 and nonpathogenic *E*. *coli* K12 [29]. Our studies show that the specific interaction between IbeA and its receptor, Vim, is the upstream signaling event, which is required for the caveolae/LR-dependent entry of *E*. *coli* K1 into HBMEC [6, 8].

The NF-κB signal transduction pathway, which is an important regulator of the innate immunity, is becoming increasingly recognized for its signaling paradigm as a good therapeutic target for molecular biomedicine including microbial infections [30]. NF-κB activation has been shown to contribute to *N*. *meningitidis* invasion of epithelium and *H*. *pylori*-induced inflammation of gastric cells. NF-κB in the CNS is activated during bacterial meningitis [8]. Recent studies show that activation of NF-κB is required for *E*. *coli*-mediated expression of ICAM-1 [31]. Our recent studies demonstrated that inhibitors of NF-κB (caffeic acid phenethyl ester), Vim (withaferin A) and LRs (filipin) were able to block IbeA^+^ *E*. *coli* K1 invasion [6,22]. Vim can form a complex with IκB, NF-κB and tubulins in the resting cells. This complex is dissociated upon the prolonged stimulation with IbeA. Vim and PSF act in concert to regulate IbeA^+^ *E*. *coli* K1-induced activation and translocation of NF-κB, and subsequently up-regulate expression of Vim, α7 nAChR and proinflammatory factors, which play an essential role in modulation of bacterial infections and inflammation associated with Ca^2+^ signaling [8,32–35].

PMN transmigration across the BBB is a hallmark feature of bacterial meningitis [31].

Transmigration of PMNs across the BBB is not only crucial for host defense against pathogens, but it may also cause significant damage to the CNS tissues, which results in devastating neurologic sequelae [19]. The adhesive interactions between transmigrating leukocytes and endothelial cells are well understood. However, the detailed mechanisms underlying the actual migration of leukocytes across the endothelium remain elusive. Recent studies have shown that blood lymphocytes and neutrophils preferentially can transmigrate across brain endothelial cells via a transcellular route [19]. Vim on both leukocytes and endothelial cells is able to form a highly dynamic anchoring structure at the site of contact between these two cell types [20]. Our previous studies showed that IbeA could play an important role in PMN transcellular migration across the BBB, which is mediated by Vim on both BMEC and leukocyte surfaces [31].

Over the past decades, the studies of neonatal bacterial meningitis caused by *E*. *coli* K1 revealed the importance and significance of IbeA contributing to bacterial adhesion/invasion, NF-κB activation and leukocyte transmigration, which are the three hallmarks (pathogenic triad) of this disease [8,19]. This occurs through interactions with host factors (e.g., Vim). One of the most challenging issues is the lack of a comprehensive understanding of the molecular basis underlying all three of the interrelated hallmark features of bacterial meningitis. Our previous findings suggest that IbeA induces all three of the hallmark features of bacterial meningitis through its receptor Vim. The underlying genetic mechanisms for these pathogenicities remain to be elucidated. In this report, using gene knockdown (cell culture) and knockout (mice) technologies, we examined how Vim contributes to the modulation of pathogen invasion, NF-κB activation and PMN recruitment/neuronal inflammation induced by IbeA^+^ *E*. *coli* K1, which is the most common gram-negative pathogen causing neonatal bacterial meningitis. The gene knockout mouse models allow us to further dissect the role of Vim in modulation of host responses that are characterized as the pathogenic triad.

## Methods and Materials

### Ethics statement

All research involving human subjects has been approved by the Institutional Review Board (IRB) of Children’s Hospital Los Angeles (CHLA). Human BMEC (HBMEC) cell line, which was immortalized by transfection with simian virus 40 large T antigen [36], was obtained from Prof. Kwang Sik Kim (Johns Hopkins University, Baltimore, MD, USA) [37]. As this cell line has been publicly available, the current research meets the criteria for Exemption 4. This cell line exhibited similar brain endothelial cell characteristics as do the primary HBMEC. These cells specifically react with Ulex europaeus lectin I (UEA I)[38], gamma-glutamyl transferase (GGT)[39], CD146 [40], and Mfsd2a [41]. The cells also exhibited the typical characteristics for the primary brain endothelial cells expressing tight junction protein ZO-1, a high transendothelial electrical resistance and polarized transport of rhodamine 123, a ligand for P-glycoprotein [42–43]. HBMEC has been shown to be the most suitable human cell line for an in vitro BBB model [36,44,45].

The animal study with mice was performed in strict accordance with the recommendations in the Guide for the Care and Use of Laboratory Animals of the National Institutes of Health. Our protocol was approved by the Institutional Animal Care and Use Committee (IACUC) of The Saban Research Institute of CHLA (Permit number: A3276-01; IACUC Protocol # 157–13). All animals were maintained in a conventional production facility and given free access to diets (Purina Picovac Irradiated Mouse Diet #5062) and water. The mice were housed in HEPA filtered ventilated racks in polycarbonate micro isolator cages equipped with an automated reverse osmosis (RO) water delivery system. All cages and bedding materials were sterilized by autoclaving before the animals were placed in the cages according to the institutional guidelines. Bedding in ventilated rodent cages is changed every 7 days or more often as necessary to keep the mice clean and dry. All mice are maintained in static micro isolator rodent cages containing 3 or more adult mice, or a female mouse with a litter, or more often as necessary to keep the animals clean and dry. The health of the animals in the conventional housing facility was ensured by a sentinel monitoring program with quarterly screens. All mouse sentinels in the housing rooms were tested to be negative throughout the course of the study. Sanitization of cages containing mice was performed according to recommendations from the Guide or inhouse standards. Inhouse standards include a complete removal of bedding weekly with a complete cage sanitization every 2 wk. The evaluation of cage density was weekly done on the basis of individual mouse weight. Animals were housed in cages that provide ample space for opportunities for normal physiologic and behavioral needs. Mice were housed on contact bedding to provide opportunity for nest building and thermal regulation. At a minimum, all animals were provided with the opportunity for visual, auditory and olfactory communication with conspecifics when possible. In addition, singly housed animals may be provided with additional environmental enrichment. Mice were provided with Nestlets (or autoclaved Kim-wipes for nude mice) as nest building materials. Mice were monitored every 24 hours by lab animal care technicians. Mouse well-being and health was assessed by observing the general appearance of animals as well as their activity prior, during, and after handling. No animals died before or during the experimental endpoint. The mice were anesthetized with Ketamine HCL (100 mg/kg) and xylazine (10 mg/kg) prior to blood collection. All efforts were made to minimize suffering. All animals received continual veterinary monitoring, and eventual euthanasia by pentabarbitol (100mg/kg) injection.

### Bacterial strains, culture conditions, plasmids and media

E44 is a rifampin-resistant strain derived from RS218 (018:K1: H7), which is a clinical isolate from the cerebrospinal fluid (CSF) of a neonate with meningitis [4]. Both RS218 and E44 have been characterized and have the same virulence phenotypes. ZD1 is an *ibeA* in-frame-deletion mutant of E44 [4]. Bacteria were cultured in brain-heart infusion (BHI) broth supplemented with ampicillin (100μg/ml), kanamycin (50 μg/ml) and/or rifampin (100 μg/ml) was used for bacterial culture if required.

### Cell culture

The immortalized HBMEC cell line [36] was routinely cultured in RPMI 1640 medium (Mediatech, Herndon, VA) containing 10% heat-inactivated fetal bovine serum, 10% Nu-serum, essential amino acids, vitamins, 1 mM sodium pyruvate, 2 mM glutamine, penicillin G (50 μg/ml) and streptomycin (100μg/ml) at 37°C in 5% CO2. *E*. *coli* invasion was performed as described previously [21].

### Chemicals and reagents

All mouse monoclonal (MMAb)/ rabbit polyclonal (RPAb) antibodies and other reagents were commercially obtained from the following sources: (A) MMAbs against vimentin (V9) (sc-6260), NF-kB (p65) (sc-109) and a RPAb against β-actin (sc-130657) from Santa Cruz Biotechnology (Santa Cruz, CA); (B) RPAbs against phospho-IKK α/β (Ser176/180) (#2697), phospho-ERK1/2 (Thr202/Tyr204) (#4370), and a MMAb against IkBa (#4814) from Cell Signaling Technology (Danvers, MA); (C) a RPAb recognizing phoshothreonine286 of CaM kinase II from Promega and a MMAb recognizing CaM kinase II from BD Biosciences; (D) a RPAb against α7 nAChR from Genescript (Piscataway, NJ) and a rat anti-mouse Ly-6G (Gr-1) antibody from eBiosciences (San Diego, CA). Blood plates and 6.5 mm diameter Transwell filters with 3 mm pore size, were obtained from BD Biosciences (San Jose, CA) as described previously [8]. Other chemicals were purchased from Sigma-Aldrich unless otherwise specified. Expression and purification of IbeA protein was carried out as described previously [6].

### Mice

Vim KO mice, previously obtained from Dr. Albee Messing at the University of Wisconsin, were originally provided by Professor Charles Babinet of the Institute Pasteur [46]. The absence of Vim has no apparent phenotypic changes in mouse reproduction and development [46]. Control mice were generated by back-breeding the KO mice with the WT strain (129/SVEV) from which they were derived for at least ten generations [47]. Mice were housed in specific pathogen-free conditions at the Animal Maintenance Facility of Children’s Hospital Los Angeles. The animals were used in transgenic breeding at 8 weeks of age for optimum reproductive performance. Male heterozygous (+/-) and female homozygous (-/-) were used in breeding. All experiments were approved by the Animal Care and Use Committee of Children’s Hospital Los Angeles.

### Hippocampal organotypic cultures (OTC)

Slices of the hippocampal formation of newborn (5- to 7-day-old) WT and Vim -/- mice were cultured as described previously [48]. Briefly, the hippocampal formations were prepared under sterile conditions, and cut transversely with a tissue chopper into slices of 400-μm thickness. After culturing 14 days, the OHCs were challenged by purified His-IbeA protein (0.1μg/ml) in culture medium for live PI staining according to the protocol (BD Biosciences) for 3 and 6 hours, and fixed by 4% PFA. The images of hippocampus with DIC and PI were acquired with immuneflorescence microscope (200 x) and analyzed by image J5.0.

### Mouse model of *E*. *coli* meningitis

Neonatal *E*. *coli* meningitis was induced as described in detail previously [6–8]. Mouse pups were randomly divided into different groups (5–6 pups/group). At 10 days of age, all animals were inoculated by i.p. injection of *E*. *coli* K1 strain E44 or ZD1 (10^5^ colony-forming units, CFU). The inoculated animals were anaesthetized with ketamine and lidocaine at 18 h post-infection. Sectioning of brain tissues and sampling of blood and CSF were carried out as described previously [23, 31]. To determine the recruitment of PMN into the CNS, CSF samples were stained with a FITC-conjugated rat anti-mouse Ly-6G (Gr-1) antibody and counted under the fluorescence microscope. The CSF concentration of albumin was measured using an ELISA kit from Bethyl laboratories (Montgomery, TX) according to the manufacturer’s instructions. To measure soluble NF-κB, CD44 or ICAM1, five μl of the CSF samples were taken and subjected to ELISA using rabbit polyclonal antibodies against NF-κB (p65), CD44 or ICAM1 from Santa Cruz Biotechnology according to the manufacturer’s instructions.

### HBMEC siRNA transfection and immunoblotting analysis

To explore the role of vimentin in NF-κB activation induced by IbeA^+^ pathogen and the relationship between vimentin and α7nAChR in HBMEC, siRNA transfection and Immunoblotting analysis were carried out as described in detail previously [6,8]. Endothelial cells were grown on 60-mm plates. Confluent HBMEC monolayers were incubated with E44, ZD1 (10^7^/ml) for 30 min or 2 hours. After the completion of incubation, total proteins of HBMECs were extracted were extracted from homogenized brain tissues with RIPA buffer supplied with 100 nM okadaic acid, 1 mM Na3VO4, 1mM PMSF as described in detail previously [7], and mixed with sodium dodecyl sulfate (SDS) buffer, heated and subjected to SDS-polyacrylamide gel electrophoresis (SDS–PAGE). Separated proteins were transferred to nitrocellulose membrane by semi-dry blotting. The membranes were blocked for 1 h with 5% milk in PBS with 0.1% Tween 20. Cytoplasm proteins in the membranes were probed with antibodies against phospho-ERK1/2 (Thr202/Tyr204) (0.2 μg/ml), phospho-IKK a/b (0.4 μg/ml), IkBa (0.2 μg/ml, Cell Signaling Technology), CamKII and phospho- CamKII (0.2 μg/ml), vimentin (V9, 0.2 μg/ml), α7 nAChR (0.1 μg/ml), and β-actin (0.1 μg/ml) for 2 hours. The membranes with nuclear proteins were incubated with antibodies against p65 (0.4 μg/ml) or β-actin (0.1 μg/ml) overnight. The washed membranes were then probed with a horseradish peroxidase (HRP)-conjugated secondary antibody for 1 h and then visualized using the same procedure as described in detail previously [7].

### Histology immunostaining

Eighteen hours after *E*. *coli* inoculation, mouse brains were harvested, fixed in 10% buffered formalin for 24 h, and embedded in paraffin. Sections with 5 mm thickness were prepared, deparaffinized with xylene and then rehydrated with graded ethanol and distilled water. Heat treatment in a microwave, blockage of endogenous peroxidase activity with 3% H_2_O_2_ and incubation with 10% goat serum were carried out as described in detail previously [7]. Anti-phospho-TAK1, anti-phospho-ERK, anti-NF-κB, or anti-phospho-CamKII (1:200 dilution) antibody was incubated with the sections overnight at 4°C. The following day, after washing, the sections were incubated with a HRP-conjugated goat anti-rabbit antibody at room temperature for 1 hour. Finally, all of the sections were stained with 3, 3-diaminobenzidine tetrahydrochloride (DAB) and then lightly counterstained with hematoxylin. Normal rabbit serum was used as a blocking agent and negative controls.

### Measurements of intracellular (Ca2+]

To examine the role of vimentin in *E*. *coli* induced calcium signaling HBMEC, determination of intracellular calcium flux was carried out as described previously [7,49–51]. Briefly, HBMEC were grown on culture dishes (MatTek, Ashland, MA) to about 40–50% confluence and then transfected with scramble, vimentin or α7nAChR siRNAs (Santa Cruz Biotechnonly) according to the manufacturer’s protocol. Transfected cells were allowed to continue growth for 48 h until 80–90% confluence. After washing with phenol-red-free HBSS and then incubation for 60 min with 4 mM Fura-2 AM and 0.04% Pluronic-127, HBMEC were then washed with phenol-red free HBSS 2 times and incubated in this buffer for an additional 20 min. Then, the intensities of fluorescence at 340 nm and 380 nm were monitored for 10 min at 4 seconds intervals. E44 or ZD1 (1×10^8^ CFU) was added at 2 min to stimulate the cells, and changes in intensities at 340 nm and 380 nm were measured. These measurements were made using a Nikon inverted microscope (Melville, NY) equipped with a Nikon Fluor 406/1.3 NA Ph4DL oil immersion objective lens as described previously [7]. Additional pieces of equipment needed for the measurements included a Hamamatsu Corp. (Bridgewater, NJ) ORCA-100 (C4742–95-12NR) 12-bit digital camera (4×4 binning mode), a Ludl Electronics Products Ltd. (Hawthorne, NY) Mac2000 XYZ stage and a focus controller. MetaMorph 4.5 (Universal Imaging Corp., Downingtown, PA) was used for the control of the imaging rig. Changes in (Ca2+] were expressed as the F340:F380 ratio, where F340 and F380 were Fura-2 fluorescence intensities obtained at 340 nm and 380 nm excitation wavelengths, respectively.

### Preparation of lipid rafts from BMEC

Lipid rafts that contained the protein caveolin were isolated using the Caveolae/Rafts Isolation kit from Sigma–Aldrich. HMBEC were transfected with scramble, vimentin or α7nAChR siRNAs (Santa Cruz Biotechnology) as described above (in the section of SiRNA transfection). HBMEC were triggered by medium (control), E44, ZD1 (5×10^7^/plate) for 2 h. After incubation, membrane lipid raft fractions were prepared as described in detail previously [6,52]. The cell lysates were prepared by passage through a 22 gauge needle 20 times, and then centrifuged at 1000 g for 10 min. The post-nuclear supernatant was adjusted to 35% of OptiPrep with 60% original stock, transferred into SW40 centrifuge tubes and overlaid with 2 ml each of OptiPrep (30, 25 and 20%) and 1 ml of lysis buffer on the top. Nine fractions (1 ml for each fraction) were collected after centrifugation at 21000 rev./min for 3 h using an SW-41 rotor. These fractions were analyzed by Western blotting with the antibodies against caveolin-1, vimentin (V9) and PSF.

### Statistical analysis

All results shown are the means+2S.D. of triplicate determinations in invasion and leukocyte transmigration assays. Microsoft Excel and the statistical package were used for the storage and analysis of raw data. Data from the animal experiments were analyzed using Software Graph Pad Prsim 5.0. ANOVA and covariates were followed by a multiple comparison test. *P*<0.05 was considered to be statistically significant.

## Results

### Vimentin deficiency protects neonatal mice from *E*. *coli* K1-induced bacterial meningitis

**Fig 1:**
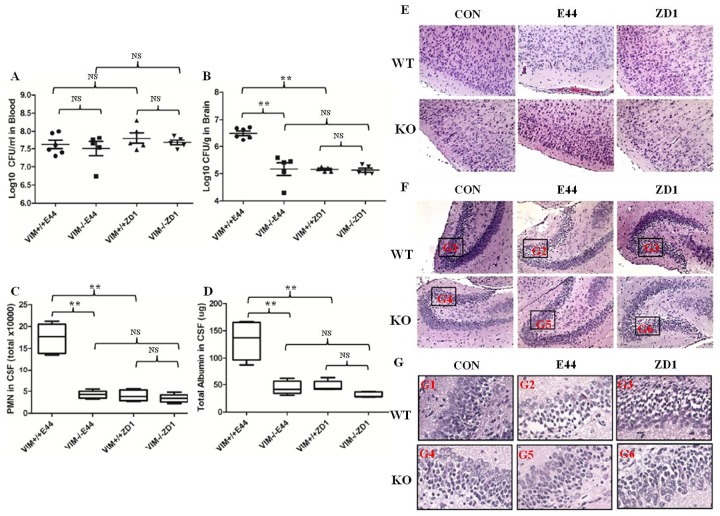
Effects of vimentin deficiency on *E*. *coli* K1-induced bacterial meningitis. A-B: Bacterial loads in blood (A) and brain (B) of WT and vimentin KO mice. C: Transmigration of PMN into the CSF of WT and KO mice. D: Albumin level in CSF of WT and KO mice. E-G: Histological examination of brain cortex (E), dentate gyrus (F) and hippocampus (G). Images (E and F) photographed at 200X magnification. Boxes in (F) show the relationship between F and G (G1–6). WT: wild-type. KO: knockout. NS: not statistically significant. Bar graphs show the means ± SD. In both invasion and PMN transmigration assays, significant differences between different groups (5–6 pups/group) are marked by asterisks (*P<0.05; **P<0.01).

In the neonatal murine model of *E*. *coli* meningitis, vimentin -/- and IbeA deletion in *E*. *coli* K1 (ZD1) did not change the bacteremia level (Fig 1A). However, the bacterial entry into brain, PMN transmigration across the BBB, and BBB permeability indicated by albumin concentration in CSF were all significantly reduced in vimentin knockout and with IbeA deletion (ZD1) as compared to wild-type mice with E44 infection (Fig 1B, 1C and 1D). Histological examination of brain sections with hematoxylin-eosin staining indicated that the blood infiltration into brain cortex (Fig 1E) and neuronal injury in the dentate gyrus of the hippocampus (Fig 1F and 1G) induced by E44 infection were both decreased in vimentin knockout by IbeA deletion (ZD1). Bacterial meningitis causes neuronal damage that predominates in the hippocampal dentate gyrus [53]. To verify the role of vimentin and IbeA in neuronal injury, the hippocampus from neonatal wildtype and vimentin knockout mice were isolated, cultured, and challenged with purified IbeA protein. Immunofluorescence examination showed the neuronal apoptosis indicated by live PI staining accumulated with IbeA stimulation in wildtype hippocampus, but not in vimentin knockout hippocampus after 3h (S1A and S1B Fig) and 6h (S1C and S1D Fig). These data demonstrated that the deficiency of vimentin protect neonatal mice from *E*. *coli* K1-induced bacterial meningitis.

### Vimentin deficiency inhibits the CNS inflammatory response

**Fig 2:**
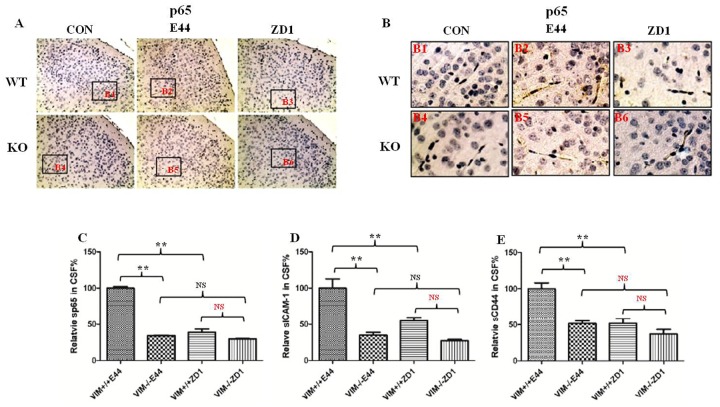
Effects of vimentin deficiency on *E*. *coli* K1-induced CNS inflammatory response. A-B: Immunehistochemical analysis of p65 expression in the brain cortex (A) and endothelial cells (B). Image (A) photographed at 200X magnification. Boxes in (A) show the relationship between A and B (B1–6). C-E: The protein level of soluble P65 (C), adhesion molecules ICAM-1 (D) and CD44 (E) in the CSF. WT and KO mice were divided into 4 groups (5–6 pups/group). Each experiment was performed three times. *P<0.05, **P<0.01.

The inflammatory response induced by *E*. *coli* K1 was also eliminated by both vimentin deficiency and IbeA deletion in this neonatal murine model of *E*. *coli* meningitis. Immunehistochemical staining showed that the expression of P65 increased by E44 infection in wildtype mice was decreased in both vimenin -/- mice and ZD1 infection in the brain cortex (Fig 2A), especially the BBB indicated by endothelial cells, which form the microvascular blood vessels (Fig 2B), as well as hippocampal CA1 (S2A Fig) and dentate gyrus (S2B Fig). The release of P65 and the adhesion molecules ICAM-1 and CD44 into CSF were all augmented by E44 infection and suppressed by both vimentin deficiency and IbeA deletion (Fig 2C, 2D and 2E), which confirmed IbeA-vimentin interaction could aggravate the inflammatory response in *E*. *coli* meningitis.

### Vimentin-regulated α7 nAChR expression in mouse brain

**Fig 3:**
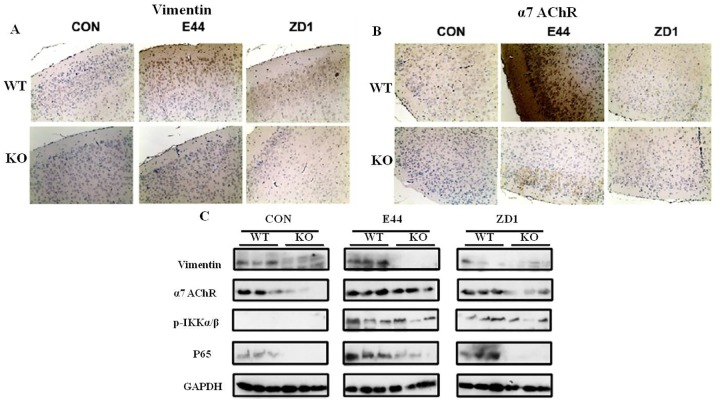
Effects of vimentin deficiency on α7 nAChR expression and NF-κB activation in mouse brain. A-B: Immunehistochemical analysis of vimentin (A) and α7 nAChR (B) expression in mouse brain cortex. Images (A and B) photographed at 200X magnification C: Western blotting analysis of vimentin, α7 nAChR, phospho-IKKα/β and P65 expression levels in mouse vascular endothelial cells.

Our previous findings indicated that vimentin-mediated initial activation of NF-κB in BMEC under *E*. *coli* K1 stimulation [8]. However, the underlying signaling pathway from vimetnin-IbeA interaction to NF-κB activation remains unclear. Since vimentin-mediated *E*. *coli* invasion is involved with calcium signaling [6], and α7 nAChR-mediated calcium signaling contributes to *E*. *coli* meningitis [7], we hypothesized that vimentin contributes to *E*. *coli* invasion and NF-κB activation via α7 nAChR-mediated calcium signaling. Immunehistochemical staining showed that vimentin expression was increased by E44 infection but not by ZD1 (the IbeA deletion mutant) in the brain cortex of wildtype mice, while there was no vimentin staining seen in vimentin knockout mice (Fig 3A). The α7 nAChR expression in the brain cortex was similarly increased by E44 infection and reduced by ZD1infection. The α7 nAChR level was largely suppressed in vimentin knockout mice under different treatments in contrast to wildtype mice (Fig 3B). Western blotting with the total brain tissues confirmed that vimentin deficiency suppressed α7 nAChR expression, and both were increased by IbeA^+^ *E*. *coli* K1 (Fig 3C). Furthermore, the level of P65 expression was enhanced by IbeA^+^ *E*. *coli* K1, and reduced in vimentin knockout (Fig 3C, lower panel).

### *E*. *coli* K1-induced intracellular calcium flux is mediated by vimentin via α7 nAChR

**Fig 4:**
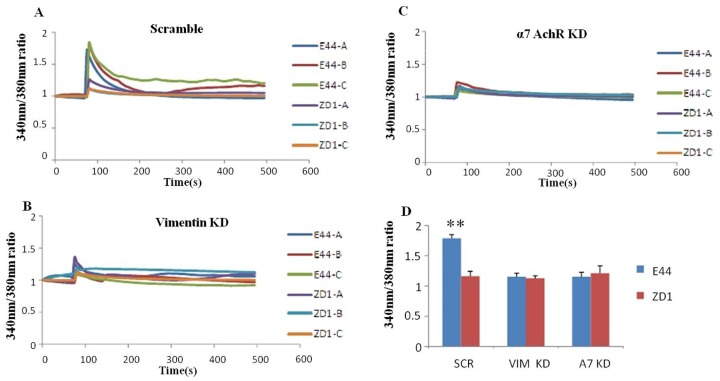
Effects of vimentin deficiency on *E*. *coli* K1-induced intracellular calcium flux. Elevation of intracellular calcium flux in HBMEC stimulated with E44 or ZD1 strains. HBMEC transfected with siRNAs of a scrambled sequence (CON), vimentin (Vim KD) and α7 nAChR (A7 KD) were loaded with Fura-2 AM as described in Methods and Materials. The monolayer was monitored for intracellular calcium flux for 10 minutes with 4 s intervals under an automated fluorescent microscope. Monolayer cells were stimulated with E44 or ZD (10^8^ CFU) at the 120 s time point. The intensity of fluorescence at 340 nm and 380 nm was measured. The ratios of intensity of fluorescence at 340 nm and 380 nm were calculated for each time interval and depicted as continuous lines in (A–C). (G) The y axis represents the ratio, and x axis represents time (s). The 340 nm/380 nm ratio changes in each treatment were calculated and subjected to statistical analysis. WT HBMEC without any pre-treatment served as a control and are defined as one-fold (1.0). (*P<0.05; **P<0.01).

Calcium ions are important for cellular signaling that is involved in the pathogenesis of bacterial infection [34]. Alpha7 nAChR has been found to play a regulatory role in the CNS inflammatory response to bacteria via its ion channel function for calcium signaling [7]. Since α7 nAChR expression was regulated by vimentin, we evaluated intracellular transient calcium flux induced by *E*. *coli* K1 using HBMEC with vimentin knockdown by siRNA. The strength of calcium flux indicated by the ratio of 340nm/380nm of Fura-2 AM was measured and calculated, showing that E44 stimulation resulted intracellular transient calcium flux immediately with a range of 0.8 fold increase on the HBMEC transfected with scrambled siRNA, while IbeA deletion (ZD1) abolished the calcium flux in these cells (Fig 4A and 4D). However, vimentin or α7 nAChR knockdown by siRNA both abolished the intracellular transient calcium flux induced by E44 and ZD1 stimulation (Fig 4B, 4C and 4D), which suggested that IbeA-vimentin meditated *E*. *coli* K1-induced intracellular calcium flux is α7 nAChR dependant.

### CaMKII activation is critical for *E*. *coli* K1-induced NF-κB activation

**Fig 5:**
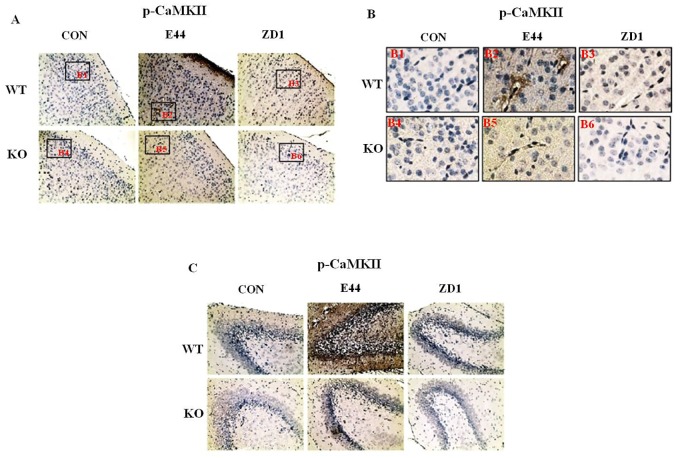
Role of vimentin in *E*. *coli* K1-induced activation of CaMKII and NF-κB. A-C: Immunehistochemical staining (DAB) was used to examine the phosphorylation level of CaMKII in the brain cortex (A), especially in the BBB indicated by endothelial cells (B), and dentate gyrus (C) of WT or vimentin KO mice infected with *E*. *coli*. The nucleus is stained as blue, and CaMKII is stained as brown. Images (A and C) photographed at 200X magnification. Boxes in (A) show the relationship between A and B (B1–6).

CaMKII has been found to relate to E. coli K1 invasion [32] and to serve as the downstream target of calcium signal through α7 nAChR ion channel. CaMKII activation could result in TAK1 and Erk1/2 phosphorylation, thus increasing IKKα/β phosphorylation and NF-κB activation. In the neonatal murine model of *E*. *coli* meningitis, immunehistochemical staining showed that E44 but not ZD1 infection could significantly enhance the phosphorylation level of CaMKII as compared to control mice in brain cortex (Fig 5A). It is especially true in the BBB indicated by endothelial cells, which form the microvascular blood vessels (Fig 5B), as well as in the hippocampal CA1 (S3 Fig) and dentate gyrus (Fig 5C) in wildtpye mice. However, vimentin knockout abolished most of the CaMKII activation in both E44 and ZD1 infected mice, which suggested vimentin mediated *E*. *coli* K1 induced CaMKII activation through calcium signaling via α7 nAChR. Furthermore, the phosphorylation levels of TAK1(S4A and S4B Fig), ERK1/2(S4C and S4D Fig), and IKK α/β (S5A and S5B Fig) enhanced by E44 infection were all found suppressed by ZD1 infection and vimentin knockout in brain cortex, as well as endothelial cells, which form the microvascular blood vessels. These results were also confirmed by in vitro studies with HBMEC, which showed that the phosphorylation levels of CaMKII, TAK1, ERK1/2, and IKK α/β and IκB degradation induced by E44 were all suppressed by ZD1 in a time course stimulation (S6 Fig). Furthermore, α7 nAChR siRNA knockdown could also abolish the phosphorylation of CaMKII, TAK1, ERK1/2, and IKK α/β and IκB degradation induced by E44. Taken together, these results suggest α7 nAChR- CaMKII play a critical role in the *E*. *coli* K1-induced NF-κB activation via a vimentin mediated calcium signaling pathway.

### Vimentin-mediated calcium signaling is lipid raft dependent

**Fig 6:**
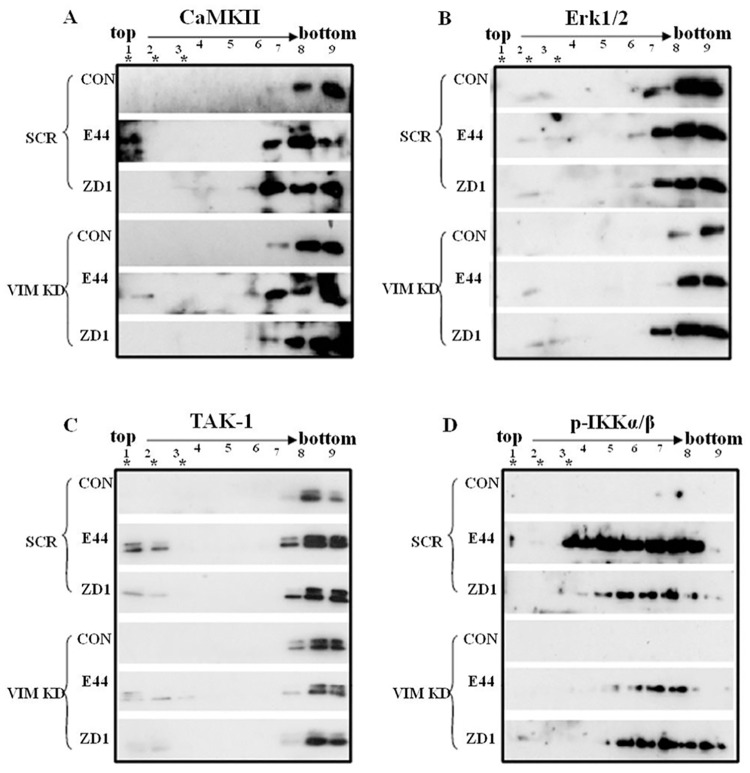
Activation of vimentin-α7 nAChR signaling cascades is lipid raft-dependent. Wild-type BMEC or vimentin KO BMEC were triggered by medium (control), E44 or ZD1 (5×10^7^/plate) for 2 h. Lipid rafts were isolated with the Caveolae/Rafts Isolation kit, which was purchased from Sigma–Aldrich. (A-D) Western blotting was used to detect distribution of CaMKII, TAK1, ERK1/2 and phospho-IKKα/β in the lipid rafts of HBMECs. Fractions 1–3 marked with an asterisk (*) consisted of caveolin-1-enriched lipid rafts. Fractions 1–9 represent the gradients from top to bottom.

In our previous studies, it has been shown that a complex containing vimentin, α7 nAChR and caveolin-1 is formed, and then recruited to caveolae upon *E*. *coli* K1 stimulation [6, 7]. It suggests that caveolin-1-enriched lipid rafts may be the functionally important signaling platform in the vimentin-α7 nAChR-NF-κB pathway. However, the underlying mechanism for this signaling cascade was still unclear. Here, we performed lipid raft fractionation analysis using HMBEC transfected with vimentin or scrambled siRNA, to dissect the relationship between the vimentin-α7 nAChR signaling cascade and lipid rafts. As shown in S7B and S7C Fig, vimentin knockdown abolished expression and recruitment of Vim and PSF to lipid raft fractions upon E44 infection, but did not change the pattern of caveolin-1 (S7A Fig). For the signaling cascade, CaMKII, Erk1/2 and TAK1 were all recruited by E44 stimulation in HBMEC transfected scramble siRNA, while ZD1 and vimentin siRNA knockdown both abolish the signaling molecules recruitment to lipid rafts (Fig 6A, 6B and 6C). The phosphorylated IKK α/β showed a trend to recruit into lipid rafts induced by E44 stimulation, while ZD1 and vimentin siRNA knockdown reduced both this trend and related phosphorylation levels (Fig 6D). These results indicate that lipid rafts play a role in vimentin-mediated NF-κB activation.

## Discussion

Bacterial invasion and translocation across of BBB are critical steps during the development of neonatal *E*. *coli* meningitis. The IbeA protein has been identified as an important virulence factor for *E*. *coli* K1 invading into BMEC, which is the major component of BBB. The specific binding of IbeA to its primary receptor vimentin is the initial signaling event, which is required for the caveolae/LRs-dependent entry of *E*. *coli* K1 into BMECs [8,21]. Subsequently, the co-receptor PSF and related signaling molecules (e.g., ERK, caveolin-1, α7 nAChR) are recruited to the LR microdomains [6–8,20]. Vimentin is expressed in abundance in mesenchymal cells such as endothelial cells [54]. It is not only involved in the invasive epithelial–mesenchymal transition phenotype of tumor cells, but also contributes to the adhesive or invasive phenotype of microbial pathogens [25,55,56]. Our previous studies indicate that vimentin is an IbeA-binding protein on the surface of HBMECs. The middle region of IbeA (271–370 residues) and the head domain of vimentin are the binding sites of IbeA–vimentin interaction [57]. Vimentin serves as the primary receptor for IbeA-induced signaling activation and *E*. *coli* K1 invasion of HBMECs [6]. Our recent findings reveal that vimentin plays an important role in NF-κB activation and translocation [12]. Using the vimentin knockout (KO) mice in this study, we find that, vimentin is the key regulator of the pathogenic triad of bacterial meningitis, IbeA-induced *E*. *coli* K1 invasion, NF-κB activation and PMN transmigration across the BBB. All three of the interrelated host responses were significantly reduced in vimentin KO (Vim-/-) mice. It has been shown that endothelial surface expression of ICAM-1 and CD44 is significantly elevated upon *E*. *coli* infection but expression of ICAM-2, VCAM-1 or E-selectin in pulmonary endothelial cells is not affected [58,59]. We also found that IbeA mediated secretion of ICAM-1, CD44 and p65 in the CSF decreased significantly in vimentin deficient mice. These data suggest that vimentin plays important role in the IbeA-induced pathogenic triad during neonatal *E*. *coli* meningitis.

Calcium-mediated cell signaling has been shown to be important for the pathogenesis of bacterial infection [34] and the biological functions of α7 nAChR [35]. Elevated intracellular transient calcium flux in HBMEC can be induced by *E*. *coli* K1 virulence factors [7]. We have previously shown that α7 nAChR participates in the modulation of meningitic *E*. *coli* K1 invasion, PMN recruitment and neuronal inflammation, and that these events are related to the intracellular transient calcium flux regulated by α7 nAChR [7]. It was unclear, however, whether there is crosstalk between vimentin and α7 nAChR in IbeA-mediated pathogenesis. In this study, we found that the expression level of α7 nAChR was significantly reduced in the brain of vimentin KO mice. The increased expression of α7 nAChR in mouse brain cortex with *E*. *coli* K1 E44 infection was also abolished by vimentin KO. Additionally, we found that the intracellular transient calcium flux in HBMEC induced by E44 stimulation was blocked by knockdown (KD) of vimentin or α7 nAChR.

**Fig 7:**
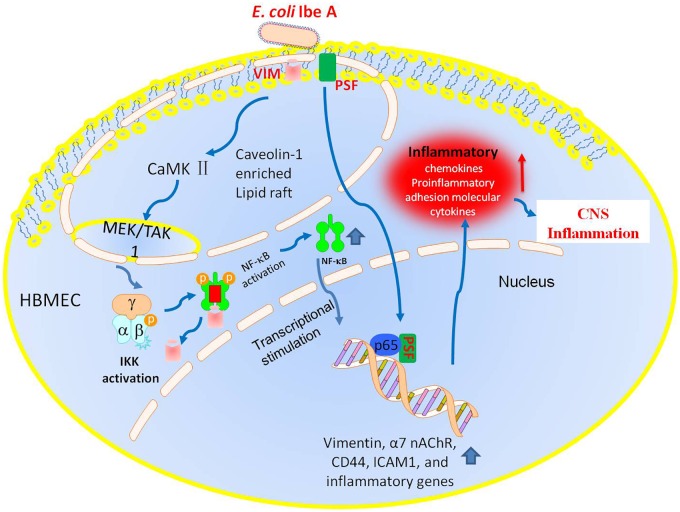
A schematic diagram of the Vim- signaling model. *E*. *coli* K1 infection triggers IbeA-dependent activation of the vimentin signaling pathway at the host cell membrane. IbeA binds to its receptor Vim and co-receptor PSF that interact with α7n nAChR through lipid rafts. These communications trigger phosphorylation of signaling proteins (e.g., Vim, TAK1), which in turn activates the nuclear factor-kappaB (NF-κB) pathways via activation of the IκB kinase (IKK) complex. NF-κB activation resulted to the nuclear translocation of NF-κB, which induces the production of cytokines, chemokines, and others proinflammatory molecules in response to bacterial stimuli.

NF-κB in the CNS is activated during bacterial meningitis [60]. It has shown that blockage of NF-κB signaling can suppress CNS inflammation and protect rat brains from inflammatory injury resulting from transient focal cerebral ischemia [61] and bacterial meningitis [62]. In our previous studies, it has been shown that CaMKII-induced phosphorylation of vimentin at Ser^82^ and the vimentin-binding domain of ERK are important for IbeA^+^ *E*. *coli* K1 invasion of BMEC [6]. In this study, we shown that vimentin KO abolished most of the CaMKII activation in E44 infected mice. The phosphorylation levels of TAK1, ERK1/2, and IKK α/β and IκB degradation representing the activation of NF-κB induced by E44 were all suppressed by CaMKII siRNA knockdown. The phosphorylation levels of CaMKII, TAK1, ERK1/2, and IKK α/β and IκB degradation induced by E44 could be inhibited by vimentin siRNA knockdown, as well as the reduced expression of α7 nAChR. Alpha7 nAChR is involved in regulation of Ca^2+^/CaMKII and /ERK1/2. So these results suggest that vimentin is critical for IbeA-mediated activation of NF-κB in HBMEC with *E*. *coli* K1infection through the vimentin-α7 nAChR singling pathway [63]. A proteomics study shows that vimentin is a raft protein present in lipid rafts of endothelial cells [64], suggesting that vimentin is required for lipid-raft-dependent cellular signaling. In our previous studies, it has been shown that *E*. *coli* K1 stimulation can induce formation of a complex containing vimentin, α7 nAChR and caveolin-1, and then recruitment of them into caveolae [6, 7]. Caveolin-1-enriched lipid rafts may be the functionally important signaling platform in the vimentin-α7 nAChR-NF-κB pathway. In this report, we further explored the underlying mechanism for this signaling cascade, and found that CaMKII, Erk1/2 and TAK1 were all recruited by E44 stimulation in HBMEC, while the *ibeA* deletion mutant ZD1 and vimentin knockdown both significantly decreased the recruitment of signaling molecules into lipid rafts. The phosphorylated IKK α/β showed a trend to recruit into lipid rafts induced by E44 stimulation, while ZD1 and vimentin knockdown reduced both the trend and its phosphorylation levels. Gene knockdown of either Vim or α7 nAChR resulted in reduced calcium signaling, suggesting that the calcium ion may serve as a molecular bridge between these two proteins through lipid rafts [65]. These results indicated that lipid raft formation is required for the vimentin-α7 nAChR-NF-κB activation pathway (Fig 7).

Taken together, all the findings in this study indicate that the triad pathogenic features of *E*. *coli* meningitis are driven by the specific interactions between the meningitic virulence factor IbeA and its primary receptor vimentin. Genetic downmodulation of both vimentin and α7 nAChR significantly blocks IbeA^+^ *E*. *coli* K1 invasion, NF-κB activation and leukocyte transmigration that occur at the BBB, which are the triad hallmark features of this disease. In this report, TAK1 has been identified as the upstream signaling molecule (kinase) to regulate NF-κB in the vimentin-α7 nAChR pathway during *E*. *coli* meningitis. The molecular and cellular mechanisms underlying crosstalk between vimentin and α7 nAChR, however, remain to be defined. Further dissecting the vimentin- α7 nAChR axis may lead to the discovery and development of novel therapeutic interventions against the triad features of bacterial meningitis.

## Database

The protein access codes in Swissprot database are listed as follows: Vimentin, Homo sapiens, P08670; Transcription factor p65 (NF-κB (p65)], Homo sapiens, Q04206; PTB-associated-splicing factor (PSF], Homo sapiens, P23246; Neuronal acetylcholine receptor subunit alpha-7 (α7 nAChR or CHRNA7], Homo sapiens, P36544; Extracellular signal-regulated kinase 1 (ERK1], Homo sapiens, P27361; Extracellular signal-regulated kinase 2 (ERK2], Homo sapiens, P28482; Inhibitor of nuclear factor kappa-B kinase subunit alpha (IKK α], Homo sapiens, O15111; Inhibitor of nuclear factor kappa-B kinase subunit beta (IKK β], Homo sapiens, O14920; Mitogen-activated protein kinase kinase kinase 7 (TAK1), Homo sapiens, O43318; Calcium/calmodulin-dependent protein kinase type II subunit alpha (CaMKII), Homo sapiens, Q9UQM7; β-actin, Homo sapiens, P60709; Sodium-dependent lysophosphatidylcholine symporter 1 (MfSD2a), Homo sapiens, Q8NA29.

## Supporting Information

S1 Fig Role of vimentin in IbeA-induced neuronal injury. After stimulation with purified IbeA protein for 3h (A) and 6h (C), the neuronal apoptosis in the hippocampus from both wildtype and vimentin knockout mouse pups (5–6 mice/group) was examined by immunofluorescence. Propidium iodide (PI) staining was used for indicating the neuronal apoptosis. The differential interference contrast (DIC) image was taken with a transmitted light photomultiplier tube detector. Images (A and C) photographed at 200X magnification. The fluorescence intensity of WT mice treated with BSA was defined as 1.0, and the relative fluorescence intensity of WT mice or vimentin KO mice was calculated as shown in B and D.
(TIF)

S2 Fig Effects of vimentin deficiency on *E*. *coli* K1-induced p65 expresion in CNS. After treatment with E44 or ZD1, the p65 expression levels in hippocampal CA1 region (A) and dentate gyrus (B) of WT mice or viemtnin KO mice were exmamined by immunohistochemistry.
(TIF)

S3 Fig Role of vimentin in *E*. *coli* K1-induced phosphorylation of CaMKII. After treatment with *E*.*coli* E44 or ZD1, the phosphorylation levels of CaMKII in the brain hippocampal CA1 region of WT and vimentin KO mice were examined by immunehistochemical DAB staining. The nucleus was stained as blue, and the phospho-CaMKII was stained as brown.
(TIF)

S4 Fig Role of vimentin in *E*. *coli* K1-induced phosphorylation of TAK1and ERK1/2. Immunohistochemical DAB staining was used to examine the phosphorylation level of TAK1 and ERK1/2 in the brain cortex (A, C), especially in the BBB indicated by endothelial cells (B, D) of WT or vimentin KO mice infected with *E*. *coli*. The nucleus was stained as blue, and phospho-TAK1 or phospho-ERK1/2 were stained as brown. Images (A and C) photographed at 200X magnification. Boxes in (A) and (C) show the relationship between A and B (B1–6), and C and D (D1-6), respectively.
(TIF)

S5 Fig Role of vimentin in *E*. *coli* K1-induced phosphorylation of IKK α/β. After inoculation with *E*.*coli* E44 or ZD1, the phosphorylation levels of IKK α/β in brain cortex (A), and endothelial cells (B) of WT and vimentin KO mice were examined by immunohistochemical staining. The nucleus was stained as blue, and phospho-IKK α/β was stained as brown. Image (A) photographed at 200X magnification. Boxes in (A) show the relationship between A and B (B1–6).
(TIF)

S6 Fig Role of vimentin and α7 nAChR in activation of CaMKII and NF-κB. HBMECs were treated with *E*. *coli* E44 and ZD1, then the phosphorylation levels of phospho-CaMKII, phospho-TAK1, phospho-Erk1/2, and phospho-IKKα/β (A) were examined by Western blotting. β-actin was used as the internal reference protein. The expression levels of vimentin KD and α7 nAChR, and phosphorylation levels of phospho-CaMKII, phospho-TAK1, phospho-Erk1/2, phospho-IKKα/β in siRNA mediated vimentin KD and α7 nAChR KD were also analyzed by Western blotting (B).
(TIF)

S7 Fig Effects of vimentin or α7 nAChR deficiency on IbeA-induced recruitment of vimentin and α7 nAChR to lipid rafts. HBMEC with gene knockdown of vimentin or α7 nAChR by transfection with siRNA were infected with the wildtype (E44) or the *ibeA* deletion (ZD1) strains of *E*. *coli* K1. The caveolin-1 protein, which is an indicator for lipid rafts (A), vimentin (B) and α7 nAChR (C) in lipid rafts of BMEC were isolated and analyzed with Western blotting. Fractions 1–3 marked with an asterisk (*) consisted of caveolin-1-enriched lipid rafts. Fractions 1–9 represent the gradients from top to bottom.
(TIF)
