# Supplementary figures and images for: Vimentin, a Novel NF-κB Regulator, Is Required for Meningitic Escherichia coli K1-Induced Pathogen Invasion and PMN Transmigration across the Blood-Brain Barrier
Source: PLoS One. 2016 Sep 22;11(9):e0162641. doi: 10.1371/journal.pone.0162641 (PMC5033352; doi:10.1371/journal.pone.0162641)

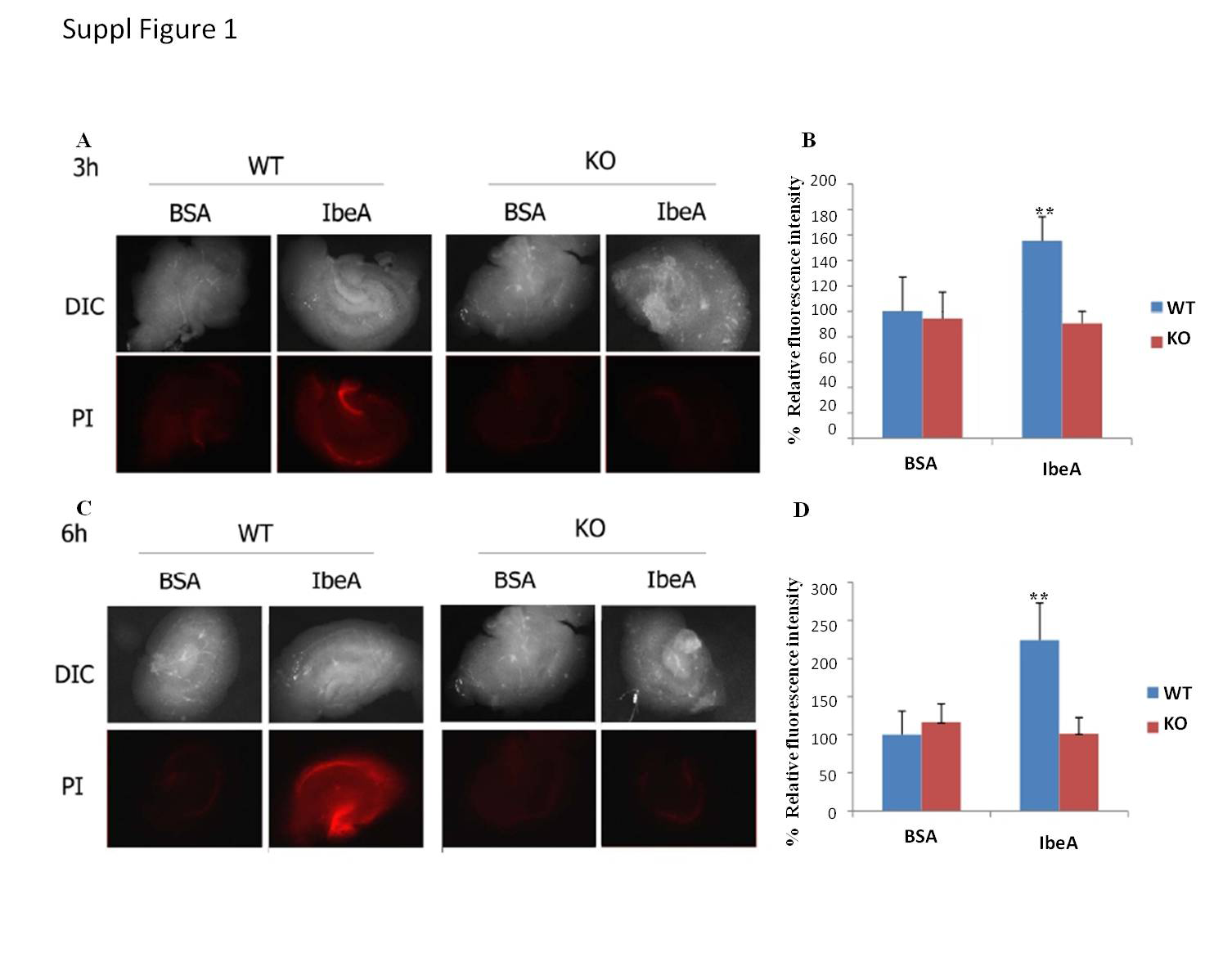

Supplement: S1 Fig — After stimulation with purified IbeA protein for 3h (A) and 6h (C), the neuronal apoptosis in the hippocampus from both wildtype and vimentin knockout mouse pups (5–6 mice/group) was examined by immunofluorescence. Propidium iodide (PI) staining was used for indicating the neuronal apoptosis. The differential interference contrast (DIC) image was taken with a transmitted light photomultiplier tube detector. Images (A and C) photographed at 200X magnification. The fluorescence intensity of WT mice treated with BSA was defined as 1.0, and the relative fluorescence intensity of WT mice or vimentin KO mice was calculated as shown in B and D. (TIF) [file pone.0162641.s001.tif]

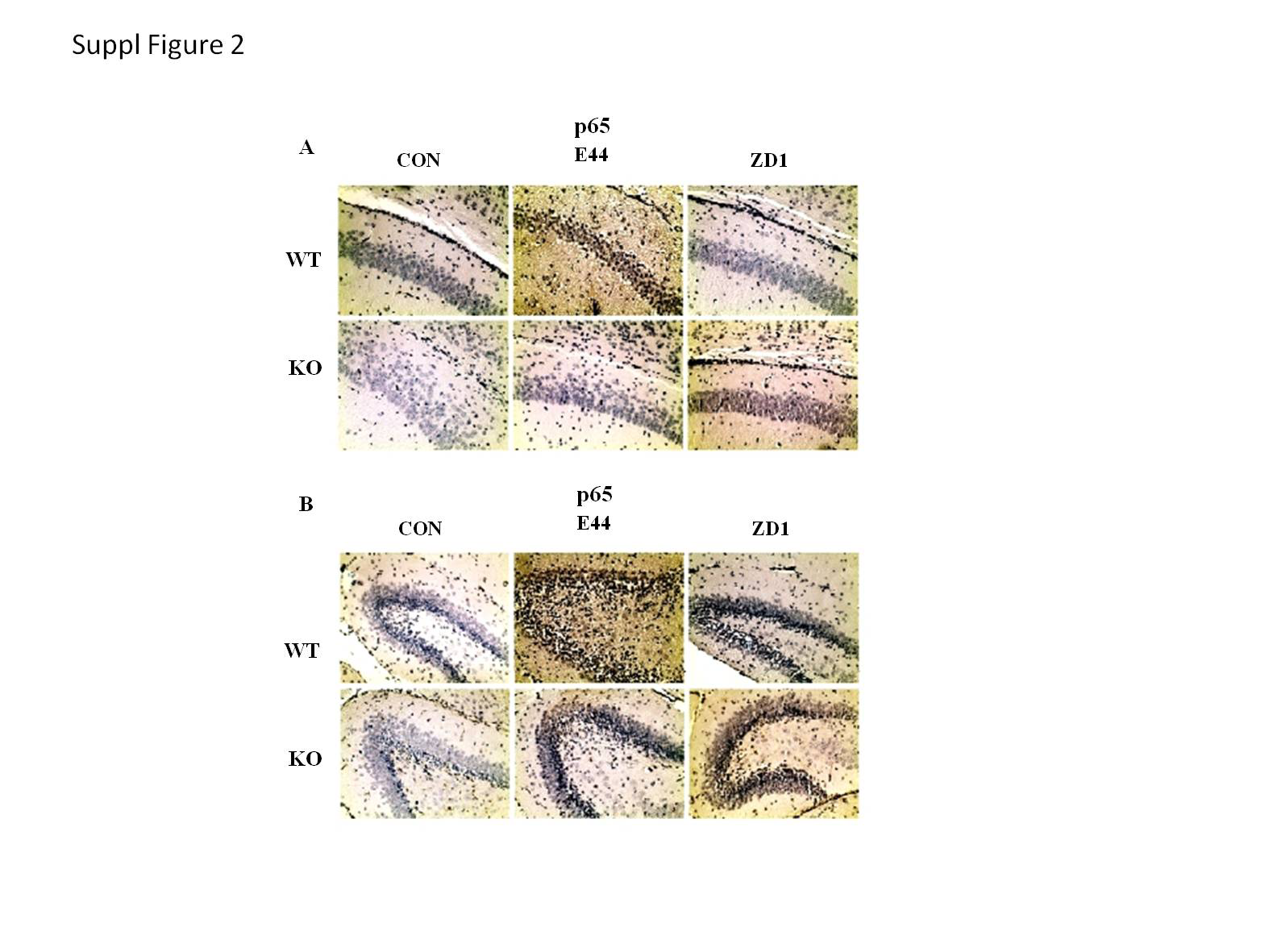

Supplement: S2 Fig — After treatment with E44 or ZD1, the p65 expression levels in hippocampal CA1 region (A) and dentate gyrus (B) of WT mice or viemtnin KO mice were exmamined by immunohistochemistry. (TIF) [file pone.0162641.s002.tif]

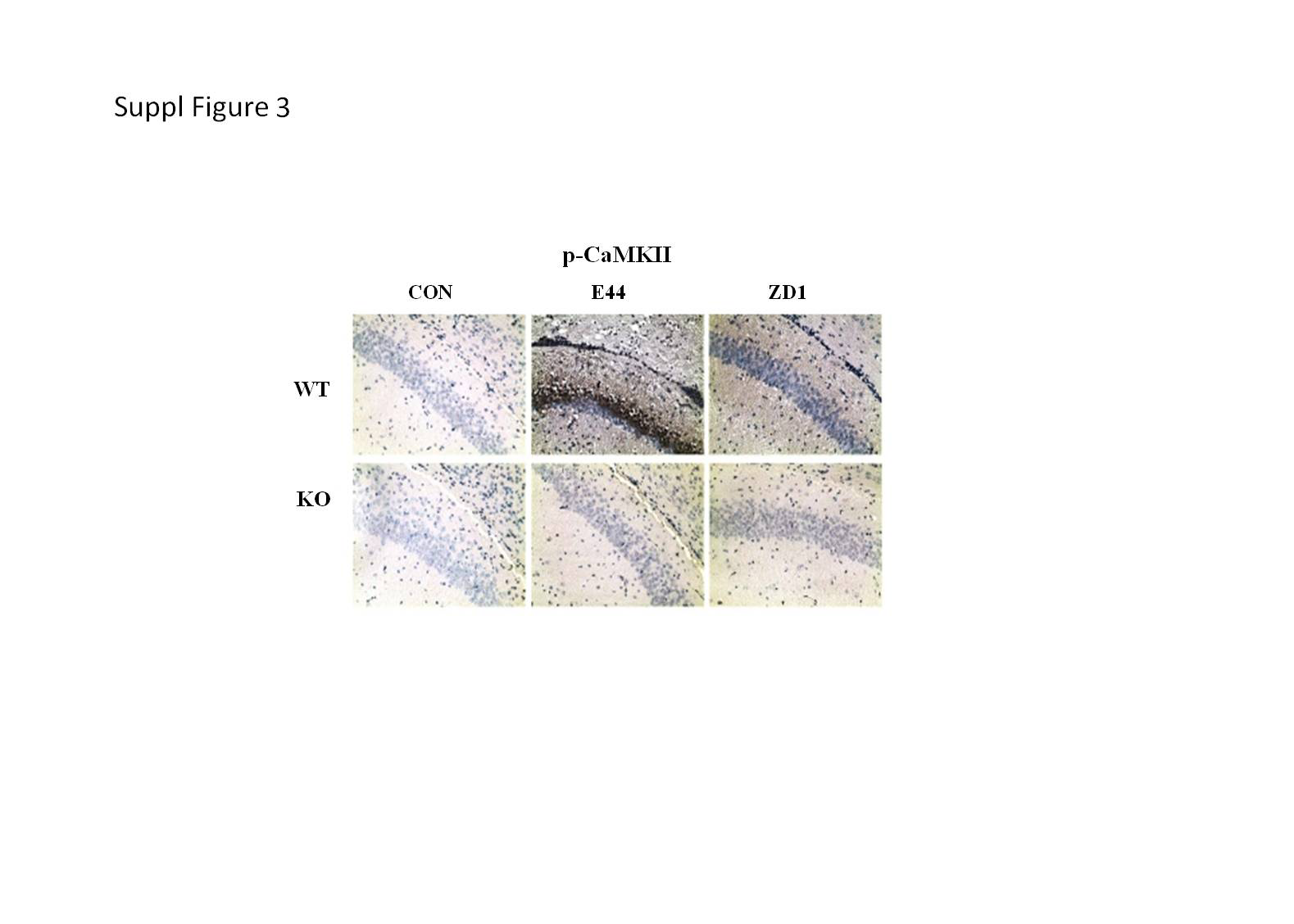

Supplement: S3 Fig — After treatment with E.coli E44 or ZD1, the phosphorylation levels of CaMKII in the brain hippocampal CA1 region of WT and vimentin KO mice were examined by immunehistochemical DAB staining. The nucleus was stained as blue, and the phospho-CaMKII was stained as brown. (TIF) [file pone.0162641.s003.tif]

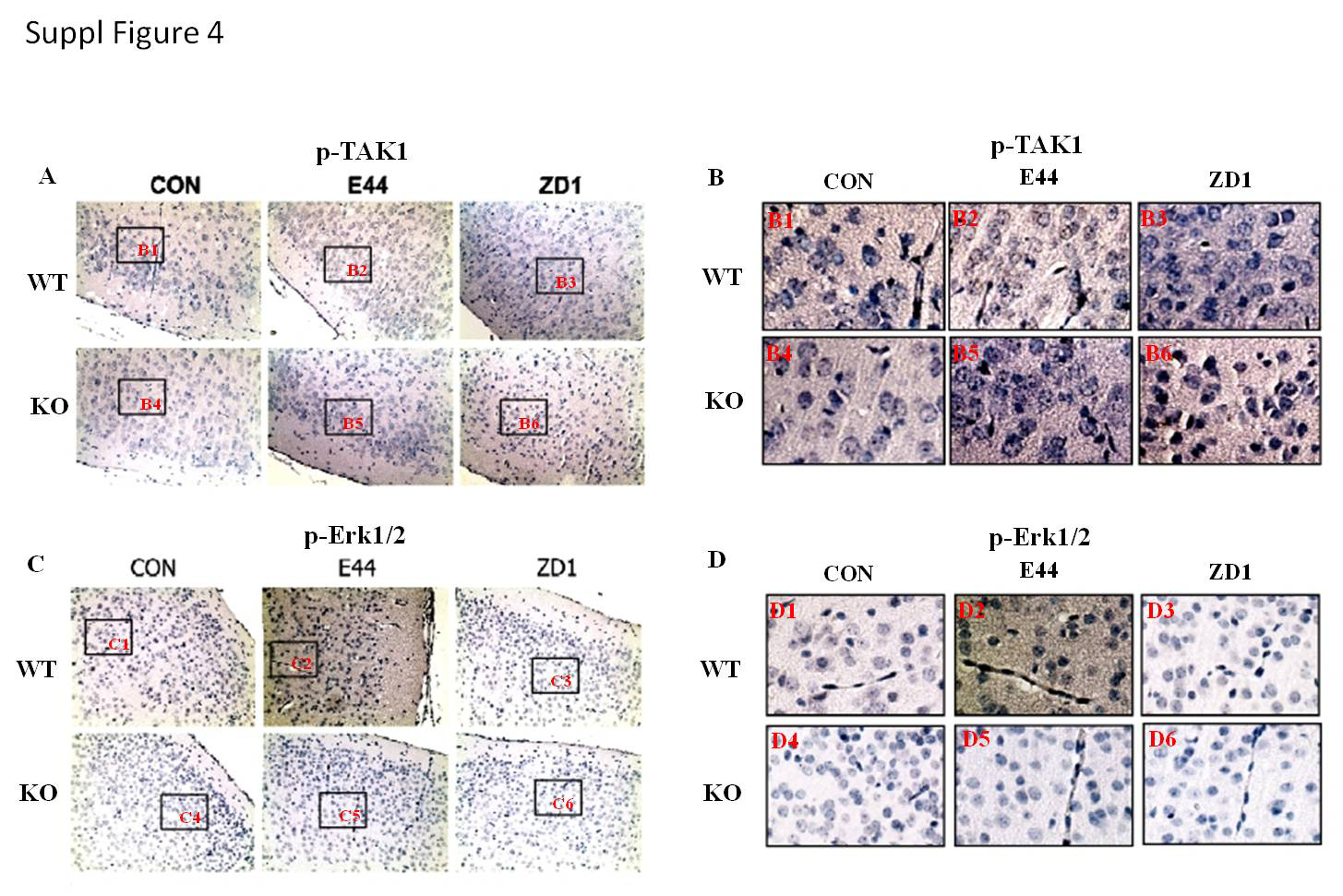

Supplement: S4 Fig — Immunohistochemical DAB staining was used to examine the phosphorylation level of TAK1 and ERK1/2 in the brain cortex (A, C), especially in the BBB indicated by endothelial cells (B, D) of WT or vimentin KO mice infected with E. coli. The nucleus was stained as blue, and phospho-TAK1 or phospho-ERK1/2 were stained as brown. Images (A and C) photographed at 200X magnification. Boxes in (A) and (C) show the relationship between A and B (B1–6), and C and D (D1-6), respectively. (TIF) [file pone.0162641.s004.tif]

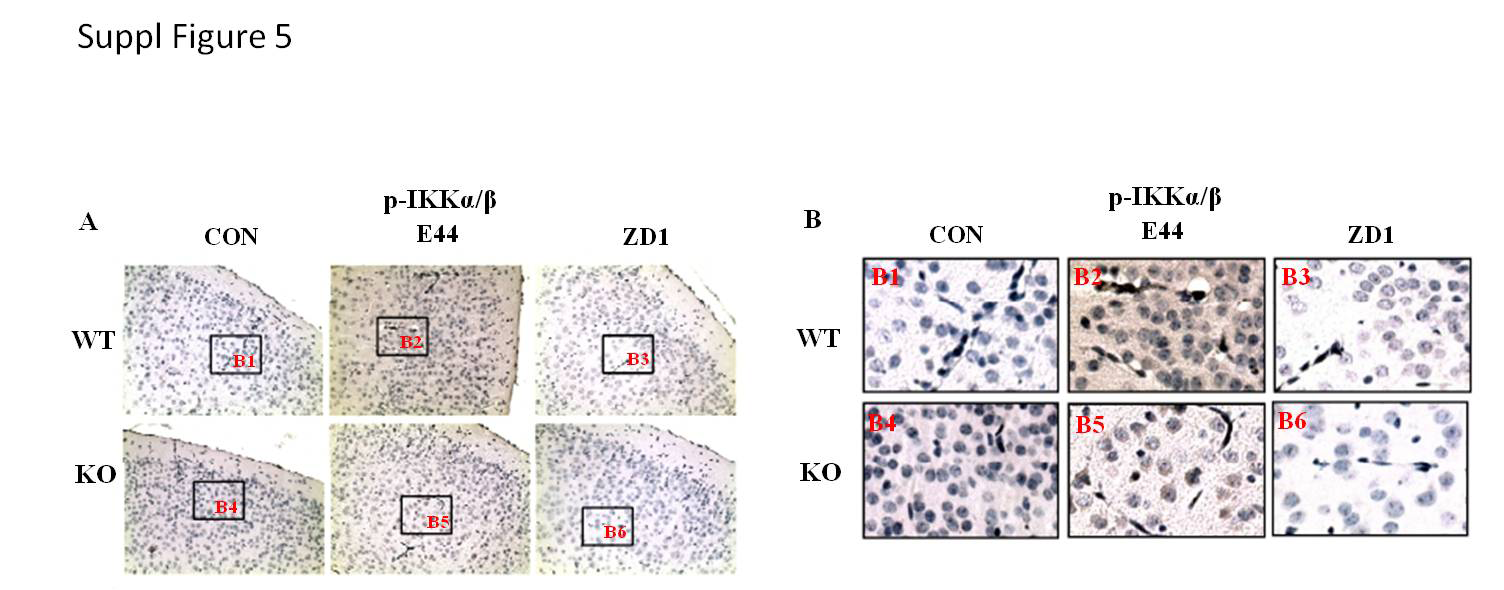

Supplement: S5 Fig — After inoculation with E.coli E44 or ZD1, the phosphorylation levels of IKK α/β in brain cortex (A), and endothelial cells (B) of WT and vimentin KO mice were examined by immunohistochemical staining. The nucleus was stained as blue, and phospho-IKK α/β was stained as brown. Image (A) photographed at 200X magnification. Boxes in (A) show the relationship between A and B (B1–6). (TIF) [file pone.0162641.s005.tif]

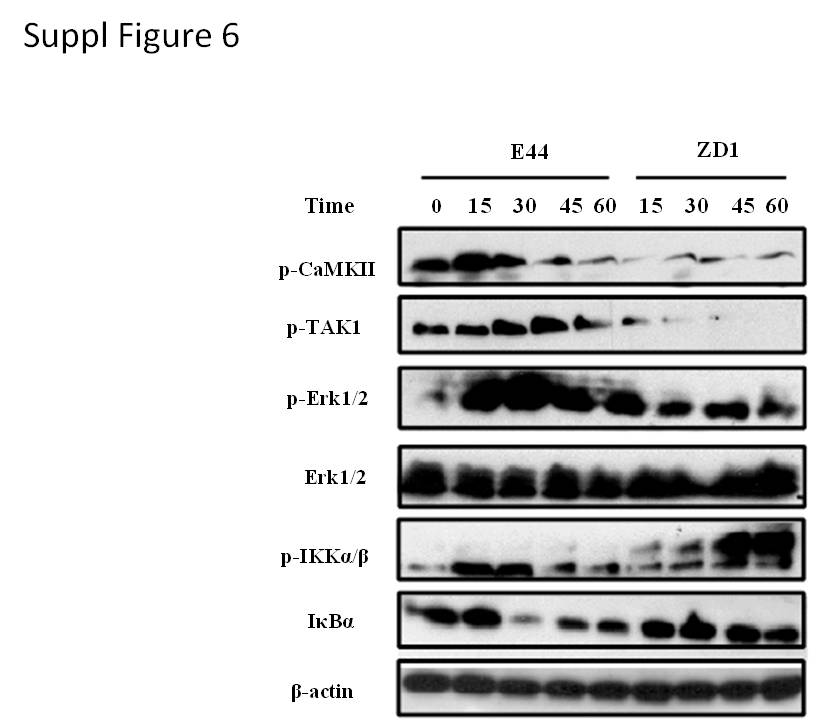

Supplement: S6 Fig — HBMECs were treated with E. coli E44 and ZD1, then the phosphorylation levels of phospho-CaMKII, phospho-TAK1, phospho-Erk1/2, and phospho-IKKα/β (A) were examined by Western blotting. β-actin was used as the internal reference protein. The expression levels of vimentin KD and α7 nAChR, and phosphorylation levels of phospho-CaMKII, phospho-TAK1, phospho-Erk1/2, phospho-IKKα/β in siRNA mediated vimentin KD and α7 nAChR KD were also analyzed by Western blotting (B). (TIF) [file pone.0162641.s006.tif]

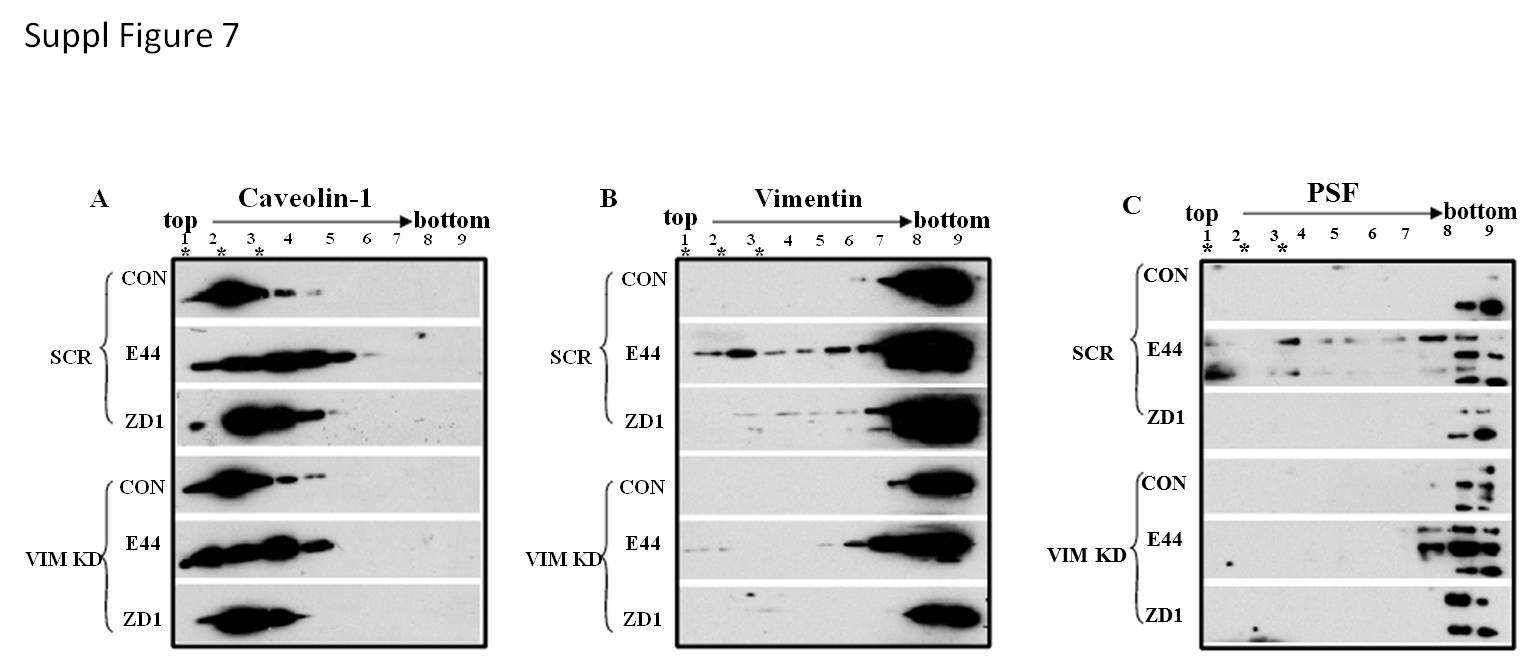

Supplement: S7 Fig — HBMEC with gene knockdown of vimentin or α7 nAChR by transfection with siRNA were infected with the wildtype (E44) or the ibeA deletion (ZD1) strains of E. coli K1. The caveolin-1 protein, which is an indicator for lipid rafts (A), vimentin (B) and α7 nAChR (C) in lipid rafts of BMEC were isolated and analyzed with Western blotting. Fractions 1–3 marked with an asterisk (*) consisted of caveolin-1-enriched lipid rafts. Fractions 1–9 represent the gradients from top to bottom. (TIF) [file pone.0162641.s007.tif]
